# Fabrication Process and Light-Trapping Performance Study of Ultrathin Silicon-Based Solar Cells with Embedded ZnO/Au Heterojunction Nanostructures

**DOI:** 10.3390/nano16030192

**Published:** 2026-01-30

**Authors:** Le Cao, Jin Zhuo, Tangyou Sun, Pengyuan Wang, Qiaonian Xu

**Affiliations:** 1 School of Information Technology, Wuwei Vocational and Technical University, Wuwei 733000, China; cl220031@wwvtu.edu.cn (L.C.); wwvtuwpy@wwvtu.edu.cn (P.W.); 2 School of Aeronautical Electrical Engineering, Zhangjiajie Aviation Industry Vocational and Technical College, Zhangjiajie 427000, China; jiangyh@advancechip.com; 3 Guangxi Key Laboratory of Precision Navigation Technology and Application, School of Information and Communication, Guilin University of Electronic Technology (GUET), Guilin 541004, China; suntangyou@guet.edu.cn

**Keywords:** zinc oxide nanostructures, AAO, silicon-based solar cells, light absorption ability

## Abstract

Owing to the excellent performance of zinc oxide materials under ultraviolet light, this paper proposes a process for fabricating ZnO/Au heterojunction nanostructures on the surface of silicon-based solar cells using anodic aluminum oxide as the template, ultimately resulting in a novel silicon-based solar cell with an embedded ZnO/Au nanostructure array. Through model optimization and analysis of the solar cells, it is found that compared with silicon-based solar cells with double grating nanostructures, silicon-based solar cells with surface silicon nanostructure arrays prepared by similar processes, and traditional planar silicon-based solar cells, the light absorption efficiency of the proposed solar cell structure is improved by 13.2%, 35.01%, and 63.78%, respectively; its short-circuit current density and power conversion efficiency reach 40 mA/cm^2^ and 20.17%, respectively. Meanwhile, this paper conducts an in-depth study on the performance enhancement mechanism, providing new insights for the fabrication of ZnO/Au heterojunction nanostructures and their applications in the field of solar cells.

## 1. Introduction

In the field of optics, nanostructures have been extensively studied due to their excellent anti-reflective capabilities, especially in the fields of solar cells and optoelectronic devices [1,2,3,4]. In 2019, Sun Tangyou and others studied the effect of nanostructures with different sidewall profiles on the photoelectric efficiency of solar cells through simulations [5]. In 2020, micro-nano structures were applied to LED devices for research, and it was found that their luminous efficiency was three times higher compared to traditional planar LEDs [6]. In recent years, template etching, water-soluble gel methods and nanoimprinting are often used to fabricate large arrays of nanostructures on the surface of optoelectronic devices, but the template method can better control the parameters of the nanostructure. Moreover, Anodic Aluminum Oxide (AAO) itself boasts advantages such as low cost, feasibility of large-area fabrication, and high process controllability. AAO is widely used as a template for nanostructure preparation and has found numerous applications in the optoelectronic field and other related areas [1,2,3,7].

Currently, in the field of solar cells, ultra-thin solar cells with high light efficiency are widely studied to reduce the cost of using solar cells [4,5]. However, the ultra-thin light-absorbing layer is not conducive to the absorption and utilization of light. To enhance the light absorption capacity of ultra-thin solar cells, numerous studies have shown that fabricating light-trapping structures on the surface of ultra-thin light-absorbing layer-based solar cells can improve their light absorption effect, thereby boosting their power generation efficiency [4,5,7]. Nonetheless, directly fabricating nanostructures on silicon-based surfaces typically results in only a modest increase in ultraviolet light absorption [5,8]. It is well known that the properties of nanostructures depend on the materials from which they are made. We have learned that the spontaneous emission of zinc oxide (ZnO) materials is enhanced under a strong electric field. At the same time, due to the quantum size effect, ZnO materials exhibit superior optical properties at the nanoscale, enabling strong absorption in the ultraviolet light range [9,10].

In this paper, the porous anodized aluminum (AAO) is used as a template, the method for forming embedded ZnO nanostructures on the surface of ultrathin silicon-based solar cells (USBS) is proposed, which uses techniques such as plasma etching, metal vapor deposition, and high-temperature oxidation annealing [11,12]. Additionally, simulation software was used to study the enhancement effect of ZnO nanostructures on the luminous efficiency of USBS. The study shows that fabricating embedded ZnO nanostructures in AAO-aligned arrays on a 2 μm-thick silicon substrate can greatly improve the photoelectric performance of ultrathin solar cells under ultraviolet light and long-wavelength light in the wavelength (λ) range of 0.2–1.2 μm. This finding provides a certain reference value for the preparation, application, and performance study of ZnO nanostructures in the field of solar cells.

## 2. Materials and Methods

### 2.1. Fabrication of USBS with ZnO Nanostructures

This study utilized a double-sided AAO template to fabricate embedded ZnO/Au nanostructures on a silicon-based surface. The preparation is divided into the following steps:

Step 1: Immerse the double-through AAO template in an acetone solution for ultrasonic cleaning for 15–20 min to remove surface organic residues, then transfer the template to the surface of the silicon wafer using the water-floating method, gently purge with nitrogen to eliminate air bubbles, and air-dry in the shade at room temperature for 1 h to ensure tight adhesion between the template and the silicon wafer.

Step 2: Adopt the plasma etching method: place the silicon-based material with the attached AAO template into a plasma etcher, evacuate the chamber until the internal pressure reaches $\leq 5\times 10^{-4}$ Pa, introduce etching gas (e.g., a mixture of ${\mathrm{SF}}_{6}$ and $O_{2}$ with a volume ratio of 5:1 and a total flow rate of 20–30 sccm), turn on the plasma etching source, and set the etching time to 30 s to 5 min. Through this step, a porous structure with an AAO-ordered arrangement can be prepared on the surface of the silicon wafer, and the specific etching time needs to be adjusted according to the required etching depth [13].

Step 3: To enhance the ultraviolet performance of the subsequently grown ZnO nanostructures. First, place the silicon substrate with a porous structure into a metal evaporation furnace, and deposit a gold (Au) layer approximately 50 nm thick via thermal evaporation (with an evaporation rate of 0.05∼0.1 nm/s and a vacuum degree of ≤1 × $10^{-4}$ Pa [14]) to modify the ZnO nanostructures. Next, place an alumina crucible containing high-purity metallic zinc (purity ≥ 99.999%) into the metal evaporation furnace, evacuate it until the vacuum degree reaches 5 × $10^{-3}$∼1 × $10^{-2}$ Pa, and then start heating [14]. When the temperature increases to approximately 460 °C and a uniform metallic luster appears on the surface of the silicon wafer, introduce a mixed gas of argon (purity ≥ 99.99%) and oxygen (purity ≥ 99.99%) to inhibit the growth of ZnO particles, where the volume ratio of Ar to $O_{2}$ is between 12:1 and 16:1 [15,16]. At this point, the evaporated zinc atoms are oxidized and deposited on the porous silicon substrate, forming ZnO nanostructures embedded in the substrate with an AAO-ordered arrangement. To avoid insufficient oxidation of the evaporated zinc atoms or the formation of large-sized ZnO particles due to agglomeration (both of which will hinder the growth of ZnO nanostructures), the following experimental conditions must be met: the evaporation rate (Revap) of metallic zinc is 1.5∼1.8 $\overset{˚}{A}$/s [16,17]; the distance between the silicon substrate and the evaporation source is not less than 20 cm (an excessively short distance tends to cause agglomeration, while an excessively long distance results in an overly low deposition rate); and the deposition temperature of the silicon substrate is 320∼360 °C [15,18]. Similarly, the specific deposition time should be determined through repeated experiments according to the target length of the nanostructures, about 5 to 10 h. The growth height of ZnO nanostructures can be calculated using the law of mass conservation [19,20,21], with the calculation formulas shown as (1)–(3) below.

(1) $$Q_{Zn}=S_{eff}\times R_{evap}\times T\times \rho_{Zn}\times Y_{evap}$$

(2) $$H_{\mathrm{ZnO}}=\frac{Q_{Zn}\times M_{ZnO}\times \;f}{S_{eff}\times \rho_{ZnO}\times M_{Zn}}\times 10^{7}$$

(3) $${H_{\mathrm{ZnO}}}_{,eff}=\frac{Q_{Zn}\times M_{ZnO}\times \;f\times \gamma \times \eta }{S_{eff}\times \rho_{ZnO}\times M_{Zn}}\times 10^{7}$$

Equation (1) calculates the total evaporation mass of metallic Zn, denoted as $Q_{Zn}$. Among them, $S_{eff}$ represents the effective area of the template, $R_{evap}$ is the evaporation rate, *T* denotes the evaporation time, $\rho_{Zn}$ stands for the density of Zn, and $Y_{evap}$ refers to the evaporation deposition yield of metallic Zn. Equations (2) and (3) calculate the theoretical height ($H_{ZnO}$) and the corrected actual height ($H_{ZnO,eff}$) of the generated oxidative nanostructures, respectively. Among them, $M_{Zn}$ and $M_{ZnO}$ represent the molar mass of Zn and ZnO, respectively; $\rho_{ZnO}$ is the density of ZnO; *f* denotes the filling factor of the nanostructures (defined as the ratio of the area occupied by the nanostructures to the effective area of the template); γ stands for the oxidation rate; η refers to the filling efficiency of the nanopores. Basic retrievable data values: $M_{Zn}$ = 65.38 g/mol, $M_{ZnO}$ = 81.38 g/mol; $\rho_{Zn}$ = 7.14 g/cm^3^, $\rho_{ZnO}$ = 5.61 g/cm^3^; γ = 0.98∼1.0 [19], η = 0.95.

Step 4: The silicon substrate embedded with ZnO/Au nanostructures is immersed in an acetone solution to remove the AAO template on its surface. After that, it is dried with nitrogen gas around 25 ℃. Subsequently, the substrate is placed in a high-temperature oxidation furnace for annealing at 300–500 °C under a pure oxygen atmosphere [22]. To prevent the ZnO nanostructures from cracking due to excessively fast heating, the heating rate is controlled at 5–10 °C per minute. It should be noted that an excessively high annealing temperature will cause sintering and agglomeration of ZnO particles, which in turn affects the final formation quality of the nanostructures.

**Figure 1:**
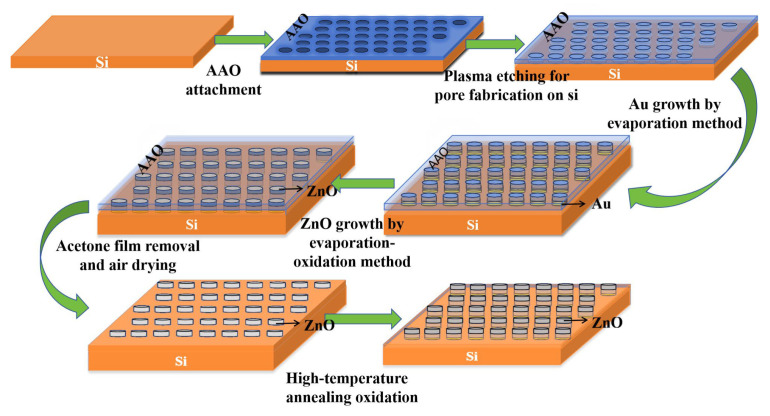
Flow chart of fabricating embedded ZnO nanostructures on the surface of USBS.

The preparation flowchart is shown in Figure 1 based on the above preparation process. In theory, an USBS with embedded ZnO/Au nanostructures can be fabricated.

### 2.2. Modeling of USBS with ZnO Nanostructures

**Figure 2:**
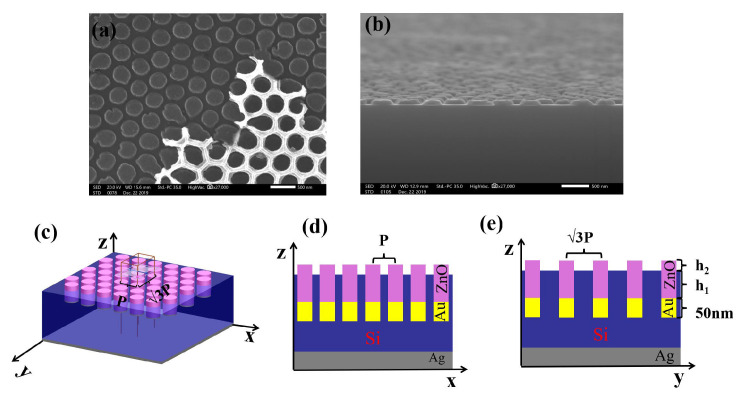
(**a**) The metal-evaporated specimen using AAO as the template. (**b**) Cross-sectional view of the xz-plane in (**a**). (**c**) Simulation model of USBS with embedded ZnO/Au nanostructures. (**d**) Cross-sectional view of the xz-plane in (**c**). (**e**) Cross-sectional view of the yz-plane in (**c**).

Optical properties are a core indicator for evaluating the power generation efficiency of solar cells. To systematically investigate the optical performance of USBS with embedded ZnO/Au nanostructures fabricated by this method, this paper establishes a simulation model by using the finite-di erence time-domain (FDTD) method [2] based on the actual structural framework determined by the experimental preparation process shown in Figure 1. A systematic study is conducted specifically on the anti-reflection performance and light absorption capacity of the cell, and the simulation model is shown in Figure 2. Figure 2a,b show the metallic columnar nanostructures prepared by metal evaporation on a silicon substrate using AAO as the template in our previous work. Furthermore, as clearly observed in Figure 2a, after growing the metal structure on the silicon-based surface via thermal evaporation, the pore structure of the AAO template remains intact, enabling it to stay attached to the surface for subsequent ZnO growth. This further demonstrates the feasibility of fabricating regular embedded ZnO/Au nanostructures on the silicon substrate via metal evaporation, and also validates the correctness of the established model.

The specific parameter settings of the model are as follows: a 2 μm-thick silicon-based material is selected as the core light absorption layer of the USBS, then a 100 nm-thick metallic silver (Ag) film is deposited on the bottom of the absorption layer, serving as a light reflection layer to promote the secondary absorption of light by the absorption layer, thereby improving light utilization efficiency. After that, embedded ZnO/Au nanostructures arranged in an orderly AAO array are constructed on the surface of the cell. Its period is P, filling factor is f, depth embedded in the silicon-based material is h_1_, and height protruding from the silicon-based surface is h_2_, and the bottom of the nanostructures is modified with a 50 nm-thick metallic Au layer.

Since the embedded ZnO/Au nanostructures fabricated via the AAO template possess a periodic structure with a symmetric triangular lattice, symmetric and asymmetric periodic boundary conditions were adopted in this study to conduct modeling and simulation of the USBS with large-area periodic ZnO/Au nanostructures constructed on its surface. Aiming to rapidly obtain accurate simulation results, the mesh precision was set to 0.005 μm [5]. During the simulation, a plane wave with a wavelength range of 0.2–1.2 μm was used to simulate the normal incidence of sunlight on the cell surface. The photocurrent density (*J_ph_*) was employed to characterize the light utilization efficiency of the solar cell, and its value can be calculated using Equation (4) [2,5]. Monitors were utilized to measure the reflectance (R) and transmittance (T) of the simulated cell, while the light absorption rate of the cell was derived from Equation (5). Considering that an Ag reflective layer was added at the bottom of the USBS absorption layer in this model, sunlight cannot penetrate the entire cell structure. Instead, it is reflected back to the absorption layer by the Ag reflective layer for secondary utilization, with only a small amount of light escaping to the external environment through the ZnO nanostructure layer. Based on this core structural characteristic, the sunlight absorption rate of the modeled cell established in this paper was ultimately calculated using Equation (6).

(4) $$J_{ph}=e\int_{200\mathrm{nm}}^{1200\mathrm{nm}}\frac{\lambda }{hc}A\left(\lambda \right)I_{AM1.5}\left(\lambda \right)d\lambda$$

(5) $$A\left(\lambda \right)=1-R\left(\lambda \right)-T\left(\lambda \right)$$

(6) $$A\left(\lambda \right)=1-R\left(\lambda \right)$$

where *A*(λ) represents the absorption of solar energy by silicon, *e* is the electron charge, *h* is the Planck constant, *c* is the speed of light in vacuum, and $I_{AM1.5}$(λ) is the incident light spectrum AM1.5.

## 3. Results and Discussion

**Figure 3:**
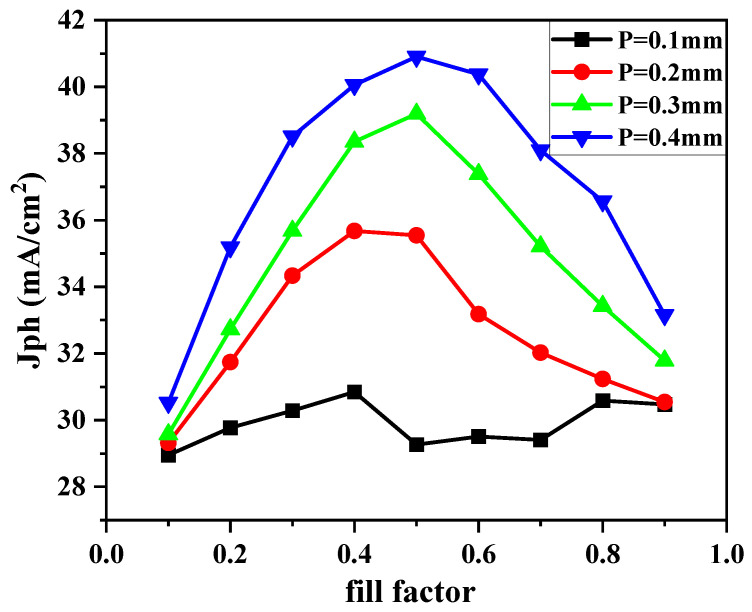
The curve of the influence of the P and *f* of ZnO nanostructures on the *J_ph_* of USBS.

To more intuitively illustrate the influence of embedded ZnO/Au nanostructures on the light absorption performance of USBS, this paper conducts simulation calculations based on the model established in Figure 2. Under the condition that the P and *f* of ZnO nanostructures are set to different parameter combinations, the *J_ph_* generated by the solar cell after light absorption is used to characterize the comprehensive performance of its light absorption capacity. A larger value of *J_ph_* indicates that the cell generates more photocurrent, has stronger light absorption capacity, and has corresponding higher photoelectric conversion efficiency. The specific simulation results are shown in Figure 3. It can be seen from the results that the light absorption capacity of the model cell is significantly enhanced with the increase in the period P; the photocurrent density *J_ph_* of the cell reaches the optimal value when the P is 0.4 μm and the *f* is 0.5.

**Figure 4:**
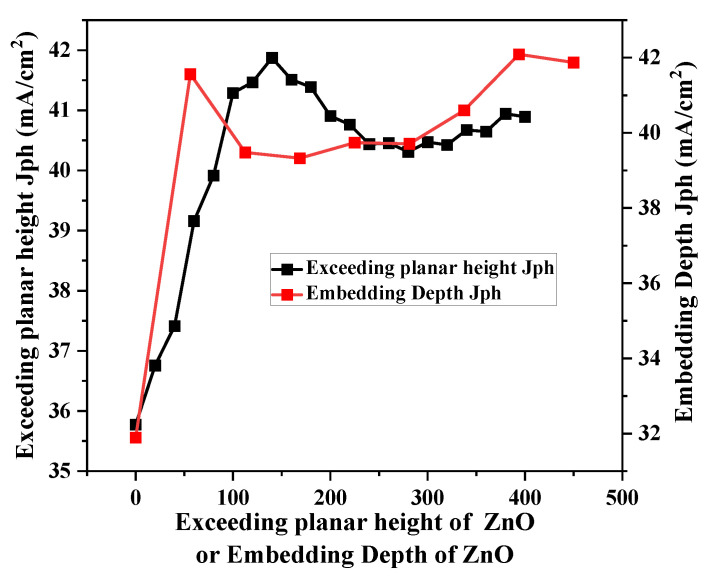
The curve of the influence of the h_1_ and h_2_ of ZnO nanostructures on the solar cell’s *J_ph_*.

To further investigate the effects of the h_1_ and h_2_ of ZnO nanostructures on the light absorption performance of solar cells, this study conducted targeted simulation research on the h_1_ and h_2_ of the nanostructures under the determined optimal structural parameters (P = 0.4 μm, *f* = 0.5). The results show that the light absorption performance of the ZnO nanostructures reaches extreme values when the h_1_ is 56 nm and 400 nm, respectively, with the light absorption effect being better at 400 nm; while the light absorption performance of the solar cell achieves the optimal level when the height of the ZnO nanostructures above the silicon substrate surface is 140 nm. The specific simulation results are shown in Figure 4.

Based on the above research results, the optimal structural parameter combination of the USBS with embedded ZnO/Au nanostructures can be determined as follows: P = 0.4 μm, *f* = 0.5, h_1_ = 400 nm, and height of the nanostructures h_2_ = 140 nm. The optimal result indicates that under the experimental conditions described earlier, the target growth height of ZnO nanostructures is h_1_ + h_2_ = 540 nm. Substituting the *f* = 0.5 and other basic retrievable data values into Formula (3) for calculation, it is found that the height of ZnO nanostructures reaches approximately 540 nm after about 7.5 h of growth. Under these parameter conditions, the solar cell can achieve optimal light absorption performance are shown in Table 1. Simulation results indicate that the *J_ph_* of this structure is improved by 13.2%, 35.01%, and 63.78 %, respectively, compared with that of the USBS with double PBN [7], the USBS with CSNP structure [23], and the planar solar cell.

As can be seen from the fabrication process of the solar cell with embedded ZnO/Au nanostructures shown in Figure 1, during the etching of nanopores on the silicon substrate surface, the AAO template is simultaneously etched, resulting in a reduction in its own thickness. To meet the requirement of etching silicon-based pores with a depth of 450 nm, two key factors need to be considered. First, the maximum fabrication thickness of large-pore ultra-thin double-through AAO templates available on the market is less than 1 μm, and the thicker the template, the more significant the error in its pore size [24], second, a 550 nm-thick double-through AAO template is selected in the experiment. After the etching of the silicon-based porous structure, the height of the subsequently grown ZnO nanostructures can be controlled within the thickness range of the remaining AAO film. This design not only conforms to the etch rate ratio characteristics of the AAO template and silicon material, but also facilitates the subsequent template removal process. In summary, the determination of the aforementioned optimal structural parameters is fully consistent with the process technology logic and practical fabrication requirements proposed in this study.

**Figure 5:**
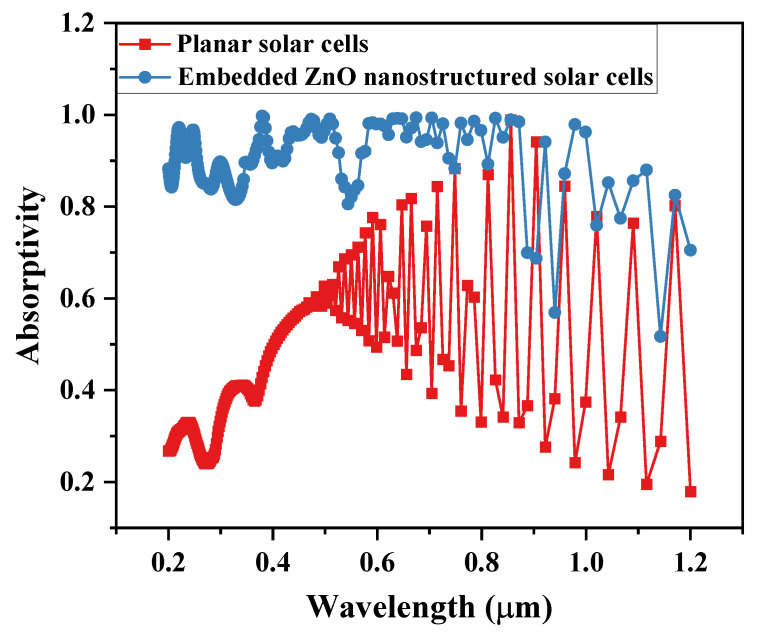
The light absorption between USBS with embedded ZnO/Au nanostructures and planar USBS.

To investigate the enhancement mechanism of the light absorption performance of USBS by ZnO nanostructures, this study conducts a simulation-based comparative analysis of the light absorption characteristics between an embedded ZnO/Au nanostructured solar cell (P = 0.4 μm, *f* = 0.5) and a planar solar cell within the wavelength range of 0.2–1.2 μm. The results are presented in Figure 5.

Analysis indicates that compared with the planar structure, the embedded ZnO/Au nanostructured solar cell exhibits significant advantages in light absorption performance. Its absorption capacity is greatly enhanced in the short wavelength range of 0.2–0.4 μm. It also maintains a considerable absorption improvement in the visible light range of 0.4–0.85 μm. More importantly, the enhanced absorption of this structure in the long wavelength range of 0.85–1.2 μm effectively compensates for the common problem of insufficient absorption in the long-wavelength region of similar nanostructured anti-reflective solar cells.

To accurately quantify the photovoltaic conversion potential of the proposed embedded ZnO/Au heterojunction structure and eliminate deviations between theoretical design and actual device performance, a three-dimensional electrical simulation model was established in this work based on the practical fabrication structure and physical characteristics of the device, enabling the precise reproduction of the device’s photoelectric response process. In the model, the Ag back electrode forms an intimate ohmic contact with the heavily doped region of the silicon substrate (with the doping concentration set to 1 × 10^20^ cm^−3^ [25]), and the high doping concentration is utilized to reduce the interfacial contact resistance. The surface Ag electrode forms a favorable ohmic contact with the 140 nm-thick ZnO film protruding from the silicon substrate surface; the ZnO layer is doped at a concentration of 4 × 10^18^ cm^−3^ [25], which effectively mitigates the contact barrier between the electrode and the ZnO layer, reduces the energy loss during carrier transport, and thus significantly enhances carrier migration and transport efficiency, laying a structural foundation for the high performance of the device [26]. To reproduce the entire process of carrier generation, transport, and recombination inside the device, targeting the intrinsic characteristic that Au nanoparticles tend to form deep recombination centers in the silicon substrate and exacerbate carrier loss, all key carrier recombination mechanisms are fully incorporated into the simulation system, including Shockley-Read-Hall (SRH) recombination, Auger recombination, and Au/Si interfacial recombination, thereby constructing a full-dimensional recombination loss model covering both the bulk phase and interfaces. Among them, the electron and hole recombination velocities at the Au/Si interface are both set to 1 × 10^5^ cm/s to simulate the characteristics of non-radiative deep carrier recombination [27,28]. In contrast, due to the differences in work function and interfacial state distribution between Au and ZnO, an asymmetric recombination velocity configuration is adopted at the Au/ZnO interface, where the electron recombination velocity is set to 5 × 10^4^ cm/s and the hole recombination velocity is adjusted to 1 × 10^5^ cm/s, which is consistent with the actual carrier recombination behavior at the heterojunction interface [28].

**Figure 6:**
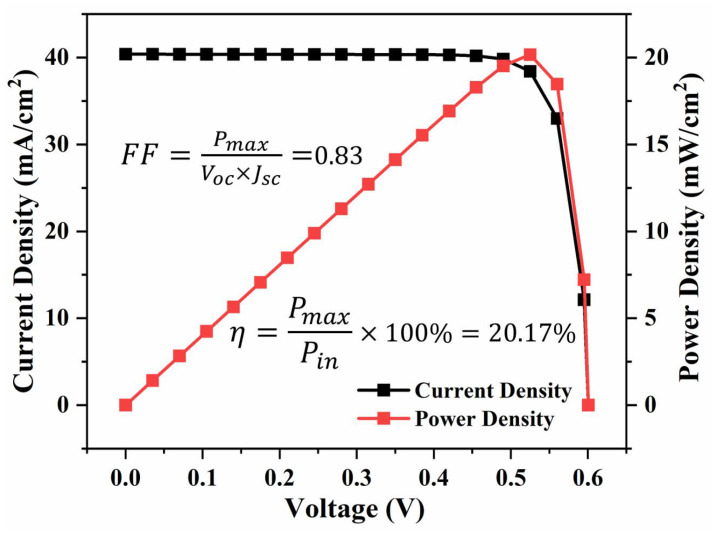
The electrical performance of USBS with embedded ZnO/Au nanostructures.

Based on the above simulation model, the core photovoltaic conversion performance parameters of the device were systematically extracted and analyzed. The results show that the short-circuit current density (*J_sc_*) is 40 mA/cm^2^, the open-circuit voltage (Voc) is 0.6 V, and the fill factor (FF) is 0.83, corresponding to a photovoltaic conversion efficiency (η) of 20.17%. The detailed curves and data distribution of the relevant simulation results are presented in Figure 6.

**Figure 7:**
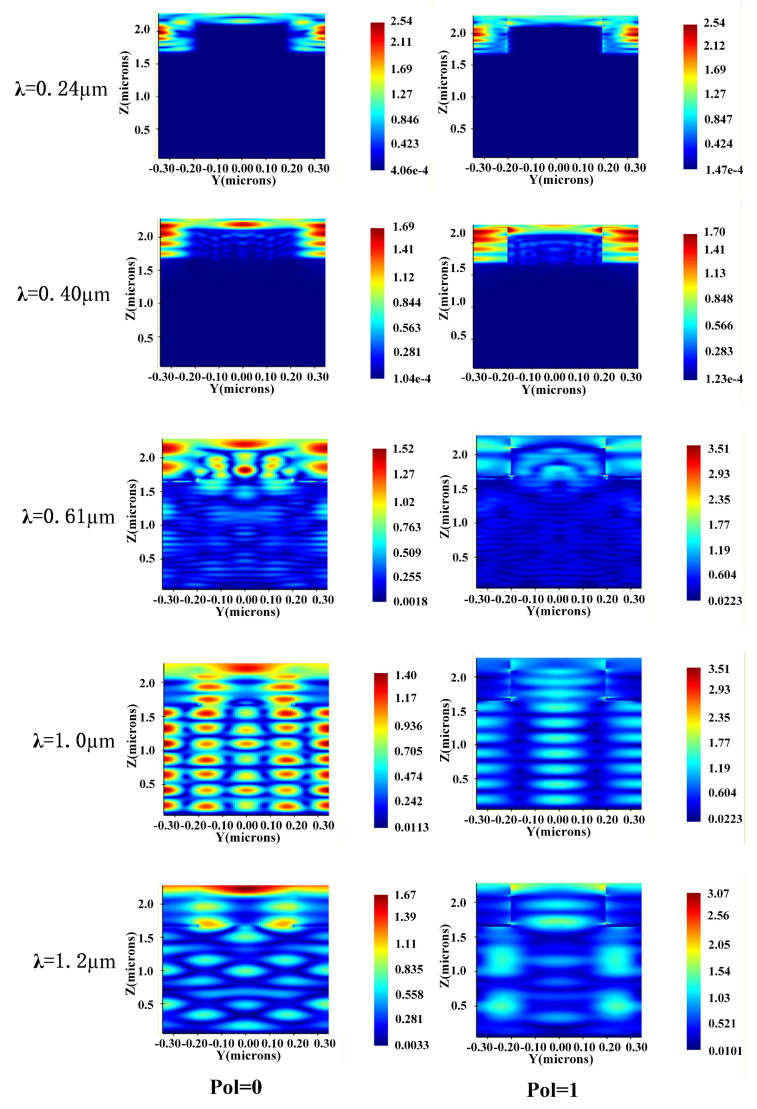
Distributions of internal optical field of USBS with embedded ZnO/Au nanostructures.

To explore the mechanism by which the embedded ZnO/Au nanostructures enhance the optoelectronic properties of solar cells, this study analyzed the optical field intensity distributions at wavelengths of 0.24 μm, 0.4 μm, 0.6 μm, and 1 μm under two polarization conditions (Pol = 0 and Pol = 1) through simulations, with the relevant results presented in Figure 7.

Observing the optical field distributions at 0.24 μm and 0.4 μm reveals that in the short-wavelength band, due to the shorter light wavelength, the optical field intensity on the solar cell surface and inside the ZnO nanostructures is significantly enhanced. This phenomenon indicates that the light scattering effect of ZnO nanostructures is particularly prominent in this band, which can convert normally incident light into obliquely incident light onto the cell surface [29,30]. Meanwhile, the ZnO nanostructures act as waveguides to confine sunlight within the structure, extending the coupling and absorption time with the silicon substrate [31]. In addition, ZnO has a band gap width of 3.22 eV [22], and the short-wavelength range falls exactly within its intrinsic absorption region [32,33]. This enables the structure to efficiently absorb a large amount of ultraviolet light and convert it into visible light, providing more photogenerated current for the solar cell and enhancing the *J_sc_* [34].

Analysis of the optical field intensity inside the cell at a wavelength of 0.6 μm shows that, based on the wavelength anti-reflection theory and effective medium theory, a large amount of visible light is effectively coupled into the cell, and the scattering effect of the nanostructures further enhances the surface coupling efficiency [31]. It is worth noting that in the medium-wavelength band, the gold nanoparticles used for modification excite surface plasmon resonance (SPR), forming a strong localized optical field in the surrounding area and significantly promoting photon absorption [35,36]. After light enters the cell, the reflective property of metallic Ag enables the secondary utilization of sunlight [35,37]. At the same time, the scattering effect of the nanostructures changes the propagation path of light, and the refractive index difference formed by the effective medium induces an optical waveguide effect inside the cell, restricting the escape of light, thereby greatly improving the absorption performance of the cell for the visible light band.

As shown in the results for the 1 μm wavelength in Figure 7, the optical field inside the cell is nearly normally incident, indicating that the effective medium effect of the zinc oxide nanostructures is more significant at this wavelength, and a large amount of sunlight is successfully coupled into the cell [30]. Furthermore, the Ag reflective layer not only prevents the escape of long-wavelength light, but also induces periodic resonance. The optical waveguide effect confines the internal optical field to form optical resonant modes and standing waves, further strengthening the secondary absorption of sunlight by the cell. Comparing the light intensity distribution at the 1.2 μm wavelength, it is found that the localized surface plasmon resonance (LSPR) and scattering effect of metallic Au can still promote optical field enhancement and absorption in the long-wavelength band [35,36]. This indicates that LSPR and scattering effects also exist for normal incidence at the 1 μm wavelength, and the coherent scattering generated at this time further increases the optical field intensity in the vertical resonant region [38] effectively improving the utilization efficiency of long-wavelength light. Meanwhile, metallic Au reduces the series resistance, enhances the carrier transport and collection capabilities, and further promotes the conversion of the *J_sc_* [39,40].

The light absorption process in the short-wavelength band (0.24–0.4 μm) is mainly completed on the surface; the optical field in the medium-wavelength band (0.4–0.85 μm) gradually intensifies toward the interior of the solar cell, forming a synergistic absorption mode of surface absorption and internal absorption; while light in the long-wavelength band (0.85–1.2 μm) achieves substantial absorption inside the solar cell. Combined with the evolutionary characteristics of optical field intensity at different wavelengths, a comparison of the differences in optical field distribution between the two polarization modes reveals that in the short-wavelength band, there is no significant difference in the light-trapping capability of the cell between Pol = 0 and Pol = 1 modes, whereas in the medium and long-wavelength bands, the light-trapping effect of the Pol = 0 polarization mode dominates. This phenomenon indicates that the optical response of zinc oxide nanostructures in the medium and long-wavelength bands has a significant polarization dependence.

## 4. Conclusions

To address the weak light absorption capacity of existing ultra-thin silicon-based solar cells in the ultraviolet and infrared regions, this paper designs a complete process for fabricating embedded ZnO/Au heterojunction nanostructures on the silicon substrate surface using AAO as the template, via techniques including plasma etching, metal oxide evaporation, and high-temperature oxidation annealing. To verify the enhancement effect of this nanostructure on the light absorption performance of solar cells, this study employs Lumerical FDTD software for modeling and parameter optimization. The results show that when the thickness of the Au layer is 50 nm, the period of the ZnO and Au nanostructures is 400 nm, the h_1_ is 400 nm and the h_2_ is 140 nm, and the device exhibits a J_*sc*_ of 40 mA/cm^−2^, an η of 20.17% and FF of 0.83. Compared with silicon-based solar cells with double grating nanostructures, silicon-based solar cells with the same-type silicon nanostructures, and traditional planar silicon-based solar cells, its light absorption efficiency is improved by 13.2%, 35.01%, and 63.78%, respectively.

## Figures and Tables

**Table 1:** Comparison of *J_ph_* between USBS with embedded ZnO/Au nanostructures and planar solar cells after parameter optimization.

Type of USBS	*J_ph_*	Comparison of Increment
USBS with ZnO/Au nanostructure	42.11 mA/cm^2^	—
USBS with double PBN structure [7]	37.2 mA/cm^2^	13.2%
USBS with CSNP structure [23]	31.19 mA/cm^2^	35.01%
planar solar cells	25.71 mA/cm^2^	63.78%

## Data Availability

No new data were created or analyzed in this study.
